# Utility and effectiveness of the Scandinavian guidelines to exclude computerized tomography scanning in mild traumatic brain injury - a prospective cohort study

**DOI:** 10.1186/s12873-018-0193-2

**Published:** 2018-11-20

**Authors:** Arurann Ananthaharan, Gunnhild Kravdal, Truls Martin Straume-Naesheim

**Affiliations:** 1 Akershus University Hospital, Lørenskog, Norway; 2 St.Olavs Hospital, Trondheim, Norway; 3 Multidisciplinary Laboratory Medicine and Medical Biochemistry, Akershus University Hospital, Lørenskog, Norway; 4 Clinic of Orthopedic Surgery, Akershus University Hospital, Lørenskog, Norway; 5 University of Oslo, Oslo, Norway

**Keywords:** Mild brain injury, Clinical assessment of CNS injury, s100b, Head injury, CT scanning, Head trauma

## Abstract

**Background:**

In 2013 the Scandinavian Neurotrauma Committee (SNC) published updated guidelines for the initial management of minimal, mild and moderate traumatic head injuries (MTHI) that included serum analysis of protein S100B as a marker for brain tissue damage. This study reviews the effectiveness of the new guidelines in a clinical setting.

**Methods:**

For all patients admitted to Akershus University Hospital (AHUS) from June 30th 2014 to December 15th 2014 with MTHI a separate form was filled in recording the time, indication and result of any S100B sampling and/or head computer tomography (CT) examinations. Data from these forms were compared to information derived from the electronic patient records for patients with MTHI and related diagnoses and data from the laboratory for all patients that had undergone the S100B analysis within the same period.

**Results:**

Five hundred seventy-five patients were identified with MTHI, S100B sampling was indicated for 223 (38.8%) patients and carried out for 188 (84.3%) of these patients. 69 (36.7%) of the patients had a negative S100B test, but a head CT scan was still performed in 31 cases despite the negative S100B test. In total the guidelines were followed for 362 of 575 patients (63.0%). 180 (31.3%) of the MTHI cases were discharged without further observation or CT examinations, including 38 (21.1%) as a direct result of S100B testing. No re-admissions or missed initial traumatic brain injuries were observed.

**Conclusion:**

The implementation of the updated SNC guidelines resulted in direct discharge of more than one third of the MTHI cases without further observation or CT examinations. One in five of these discharges was a direct result of S100B testing. However, compliance to the guidelines were poor and the guidelines were only followed in 40%. While this study showed benefits of implementing SNC guidelines to reduce the number of CT scans, additional training is needed for optimal use.

## Background

Minimal, mild and moderate head injuries (MTHI) as defined by the Scandinavian Neurotrauma Committee (SNC) constitute up to 95% of all traumatic head injuries [1], with just a small portion of these diagnosed with intracranial pathology on computed tomography (CT) scanning, and even less requiring neurosurgical intervention [2, 3].

In 2000 the SNC published their first guidelines for initial management of MTHI on behalf of the Scandinavian Neurosurgical Society. These guidelines recommended using CT examinations rather than observation to rule out more severe injuries in these patients [4].

**Fig. 1:**
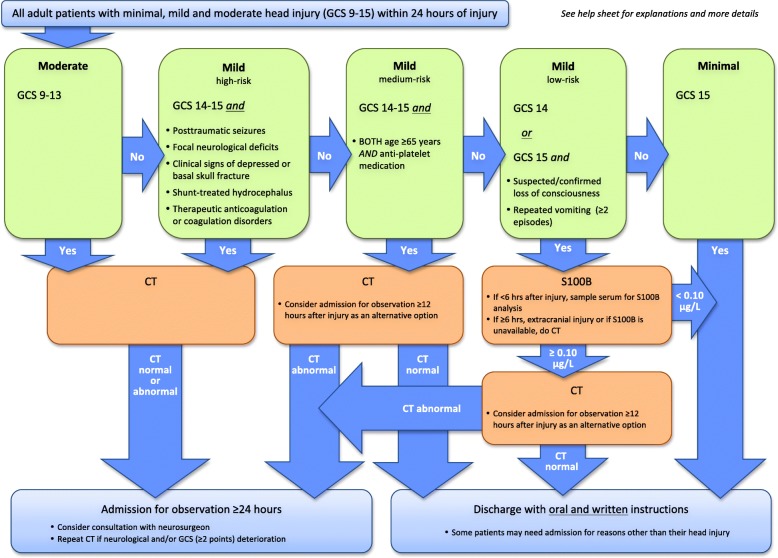
Flowchart of the updated Scandinavian guidelines for acute management of adult patients with minimal, mild or moderate head trauma. Permission to include the flowchart granted from the copyright holders [12]

The Nordic Radiation Protection co-operation wrote in 2012 that they were concerned over the rapidly increasing amount of CT examinations in Norway [5]. In 2012, Norway had the highest amount of CT examinations among the Nordic countries. Head CT examinations did also increase partly due to the new guidelines, from 20 per 1000 inhabitants in 1993 to 40 per 1000 inhabitants in 2002 [6]. The awareness of this increase led to a strong effort to reduce the use of CT examinations, not only as a result of the costs [7] but also because of the exposure to radiation. As a part of the strategy to achieve this, multiple studies have been exploring the possibilities to find a serum marker for intracranial damage where protein S100B has proved to be the most reliable [8–11]. As result, the SNC published a new update to the Scandinavian guidelines for acute management of MTHI in adult patients in 2013 [12]. These guidelines included measurement of protein S100B levels in serum with the purpose of identifying patients with a low risk of having intracranial injuries where a head CT examination and/or hospital observation was not indicated (Fig. 1).

The updated Scandinavian guidelines for initial management of MTHI including assessment of protein S100B was included in the diagnosis of head traumas at Akershus University Hospital (AHUS) in June 2014. A published validation study of the guidelines performed in New York, US, found that up to one third of the head CT scans could have been safely avoided if the guidelines had been applied [13]. The main object for our study was therefore to evaluate the clinical use of the guidelines and assess whether the guideline including the S100B test fulfils its purposes in an everyday clinical setting.

## Methods

The study included all adult patients (≥18 years) who were admitted at the Emergency Department at AHUS with MTHI and suspected brain injury for the 5.5 months between June 30th 2014 and December 12th 2014. As part of the implementation process of the new guidelines a separate form was designed to follow each patient at admission that included information of the study and a flowchart including the new guidelines. On this form the following data was noted:

- Glasgow Coma Scale (GCS) on admission (GCS at scene was included in the clincal decisions as well)
- The time of injury
- The time the serum was sampled
- Whether the serum was sampled less than 6 h after the time of injury or not
- S100B under or above the cutoff at 0.10 μg/L
- Whether a head CT scan was performed or not

After the implementation period of 6 months a retrospective search was conducted based on the hospital’s electronic patient records (EPR) to verify the information in the forms and to identify patients with related diagnoses who were not included in the forms. Records from patients with the following diagnoses were examined:

S06.0 Commotio cerebri.

S06.1 Traumatic brain oedema.

S062 Diffuse brain injury (contusion INA, laceration INA).

S06.3 Focal brain injury (contusion, laceration, traumatic intracerebral haemorrhage).

S06.4 Epidural haemorrhage.

S06.5 Traumatic subdural haemorrhage.

S06.6 Traumatic subarachnoid haemorrhage.

S068 Other specified intracranial injuries (traumatic cerebellar haemorrhage, intracranial INA).

S06.9 Unspecified intracranial injury (brain injury INA).

In addition, lists were collected from the laboratory containing all the patients who had a S100B test performed during this period, as low-risk mild head injury was the only indication for this sample at our hospital at that time. The S100B samples were analysed using the Elecsys S100 (Roche Diagnostics, Mannheim, Germany).

The forms from the study, EPR information, the results from the laboratory and the head CT examinations were then compared to get an understanding of the compliance to the updated guidelines and whether the implementation of the guidelines including S100B did reduce the number of head CT examinations. Compliance with the guidelines was estimated by dividing the number of patients who were treated according to the guidelines by the total number of patients with head trauma in this period.

The EPR were analysed throughout the observation period to identify death, missed bleedings and poor outcomes. These records are considered as a reliable source to identify re-admissions and deaths as the Norwegian health system is based on public hospitals that are responsible for specified geographic areas and with minimum patient leakage to other hospitals for the acute cases and patients attending private hospitals for emergency care is extremely seldom in Norway.

Turnaround time for S100B was calculated based on the time given when the test was ordered, which was automatically registered in the electronic patient record and the time the results were available for the doctors to view, which was automatically registered in the laboratory information system.

## Results

**Fig. 2:**
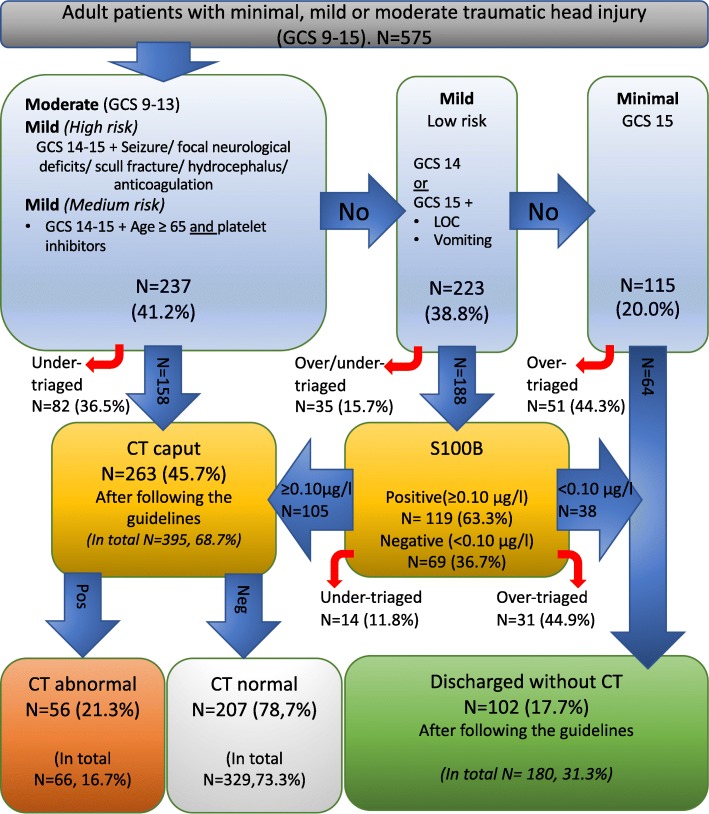
Flowchart of the results from the first 6 months with the new guidelines at Akershus University Hospital. Red arrows: Failure to perform an indicated assessment according to the guidelines (undertriage) or an assessment performed outside the indicated guidelines (overtriage). All numbers marked «In total» refers to all patients including the cases where the guidelines were not followed

A total of 575 patients were registered with MTHI in the Emergency Department during these 6 months. The mean age for all these patients was 51.2 years (SD 22.5), but the “direct CT” group (“Moderate to Mild – *medium risk*” in Fig. 1) was significantly older with a median age of 68.0 (interquartile range (IQR) 41.5–81.5) compared to the two other groups (“Mild – *low risk”*: 43.0, IQR 26.0–60.0 and “Minimal”: 39.0, IQR: 28.0–49.0, *p* < 0.005, Non parametric Kruskal-Wallis test). Adherence to the SNC guidelines and patient flow for the first 6 months of using the new guidelines at our hospital is shown in Fig. 2.

A S100B sample was indicated for 223 patients (38.8%) but performed for 188 (84.3%). Among the 188 patients 69 (36.7%) patients had a negative S100B test, defined as values less than 0.10 μg/L*.* Still, in 31 of these patients a CT examination was performed, so the number of CT examinations avoided based on the S100B screening were therefore 38 (8.2% of all MTHI patients potentially requiring a head CT (“Moderate” to “mild” groups), 21.1% of all directly discharged patients). There was no intracranial pathology found in any of the 31 patients where a CT scan was performed despite a negative S100B result.

When analysing the deviations from the guidelines, failure to perform an indicated assessment according to the guidelines (undertriage) was the case for a total of 104 (18.0%) head trauma patients, while assessment performed outside the indicated guidelines (overtriage), was done in 109 (18.9%) of the cases, resulting in a total of 213 cases where the guidelines were not followed. The corresponding calculation for the compliance to the guidelines shows that the guidelines were followed in 362 patients (63.0%, Fig. 2*)*. The further details of these assessment deviations from the guideline are summarised in Table 1.

**Table 1:** Overview of the patients where the guidelines were not followed

	N	Details	Overall Outcome
*Undertriage*	104 (18.0%)		
Discharged despite indication for direct CT	7 (6.7%)	5 < age 65 on platelets inhibitors	In total 38 patients (6.6%) did not receive recommended assessments (Clinically undertriaged). No negative outcomes were recorded in EPR
		2 on novel oral anticoagulants (NOAC)	
S100B despite indication for direct CT	75 (72.1%)	*52 positive S100B and 43 of these underwent a CT scan.*	
		*23 negative S100B, but CT was performed for 21 cases.*	
Discharged despite indication for S100B	8 (7.7%)	*Guideline deviated based on clinical assessment.*	
Discharged despite positive S100B	14 (13.5%)	*Guideline deviated based on clinical assessment*	
*Overtriage*	109 (18.9%)		
Direct CT despite indication for S100B	27 (24.8%)	*In situations where S100B is not available, this would be correct triaging. But S100B was available in the whole study period.*	In total minimum unnecessary CT scans = 40 (10.1%*) Unnecessary S100B samples = 47 (21.3%**)
CT despite negative S100B (Indicated samples)	31 (28.4%)	*All CT’s were negative*	
S100B despite indication for direct discharge	47 (43.1%)	*25 Positive S100B, resulting in 9 CT scans. All negative*	
		*22 negative S100B samples*	

The percentages are reported as a percentage of all patients with MTHI, or as a percentage of either the over- or the undertriage groups. *Percentage of all CT scans performed. **Percentage of all S100B samples performed

A total of 180 (31.3%) patients were discharged directly after primary assessment +/− S100B sampling without further CT scan or observation, but for 78 (43.3%) of these patients the guidelines were not followed. No re-admissions, missed intracerebral bleedings or deaths were noted for this group during the 6 months observation period.

The median time from ordering till the S100B test results were received was 1 h 56 min (95% CI 1 h 11 min – 3 h 44 min). Data collected from the imaging department at AHUS show that the mean time from the requisition of a CT examination until the CT study was finished and ready for examination by the ordering doctor was 1 h 36 min (waiting for the radiologist’s description often delays the process further).

## Discussion

The implementation of the updated Scandinavian guidelines for acute management of adult patients with MTHI at the Emergency Department at AHUS resulted in 31.3% of the cases being discharged directly after the primary assessment without further observation or CT examinations. Correct use of S100B contributed to approximately 20% of the total number of discharges. These findings are more or less equivalent to the values predicted from a previous retrospective study on MTHI patients [13]. Interestingly, in the clinical setting of this current study this predicted result was reached although the recommended guidelines were *not* followed for almost 40% of the MTHI cases. However, no readmissions, missed intracerebral bleedings or deaths were recorded for any of the cases during the 6 months follow-up period.

### Compliance to the new guidelines

The effects of the introduction of S100B in a clinical setting does – in addition to the test’s properties – depend on the compliance to these new guidelines. The 63.0% compliance to the guidelines found in this study is somewhat better than the 51% compliance to the first guidelines published by SNC in 2000 found in a previous study from a different Norwegian university hospital [14]. This study was followed by an education program that instructed the doctors about these guidelines which was found to increase the compliance to 63% [15]. The ability to make decisions according to the guidelines are better in this study conducted at AHUS than reported in the first study, and equally as good as the results after the education program in the second study.

Reasons for deviating from a guideline could be based on lack of knowledge about the guidelines, but also based on clinical judgment and experience. Guidelines are just guidelines, they are not strict rules of management, and the clinical judgment can therefore overrule them [12]. However, when looking at the details for cases where the guidelines were not followed (Table 1) it is clear that the vast majority of the decisions to deviate from the guidelines were within the groups where a CT scan was either *“evidently”* indicated or *“most likely not”* indicated where a “just in case” S100B-sample was performed. The most evident were the 75 S100B samples from the patients where a head CT was directly indicated and 65 of these patients (86.7%) went on to have a head CT irrespective of the S100B result. Another possible reason for overtriaging was that the patients with MTHI were considered to be in the second highest urgency level on triage, and the S100B test was ordered by default by the nurses when the patient was entering the ED to save time. This overuse of the S100B tests have little effect on the clinical value of the test but are more interesting from a financial point of view. Simultaneously the number of clinically undertriaged patients who theoretically could have an undetected intracranial injury (6.6%, Table 1) is comparable to the numbers reported for the previous guidelines, where undertriaging was observed in 7% of the cases [15].

Effective patient turnover is always in focus in a busy emergency department and such diagnostic impatience might trigger doctors to try to make shortcuts with regard to the guidelines. This was most likely the case for many of the patients in this study where a direct CT examination was indicated, but a S100B sample was ordered as well while they were waiting for an CT examination. On the other hand, since the patient’s stay at the hospital could be extended 2 h because of the S100B test, there are also several cases where a CT examination is carried out even though the patient is only indicated for the S100B test. Thus, it is evident that there is both clinical and administrative overruling of the guidelines in addition to the non-compliance due to lack of knowledge.

The two previously mentioned studies by Heskestad et al. [14, 15] indicated that deviations from the guidelines were mostly overtriaging with an excessive use of CT examinations and unnecessary hospital admissions. This is also the case in the study conducted at AHUS, but the 18.9% overtriage found in this study is lower than the results obtained in Heskestad’s study from 2012 where this was the case for 30% of the patients [15].

The over- and undertriaging seem to occur in random and no relation is identified to other factors such as certain clinicians, age of the patient, mechanism of injury, comorbidities, certain time of the day or day of the week.

### The clinical usefulness of S100B testing

In the updated guidelines it is estimated that the S100B test could replace the CT examination in approximately 30% of the patients based on several studies that have shown a negative predictive value of 97–100% and specificity above 30% with a cutoff value at 0.10 μg/L [9, 11, 13, 16, 17]. Although we were able to reproduce the same ratio of negative samples as previously described in the literature [13], the negative S100B result was ignored for almost 50% of the cases. And if looking at the spectrum of the MTHI patients admitted to AHUS in the study period that would potentially require a head CT, S100B sampling changed the pathway for only 8.2% cases compared to the old guidelines published in 2000 [4]. Optimal compliance to the guidelines would double the percentage, but still for our MTHI group, S100B sampling would only be indicated in one in three patients and then useful for only one in three of those. As a result, doctors might find the guideline less useful and this could also be an explanation for the low adherence to the guidelines and the blood sample result.

However, S100B has better sensitivity, negative predictive value and specificity than D-dimer, which is widely used in ruling out thromboembolism [9]. A negative S100B test makes it safe to send the patient home when a clinical evaluation cannot rule out brain injury and the patients are spared for a significant amount of radiation when a CT scan is avoided. This is particularly important for the younger age groups. One of the more frequent indications for direct CT within guidelines are ages ≥65 and anticoagulants and consequently there is a potentially larger percentage of the young MTHI patients that are within the group where S100B is indicated.

In this current study, over 40% of the patients were within the group where a head CT was directly indicated. This is about twice as many as reported in a previous study assessing the potential impact of the new SNC guidelines [13], and this shift towards the left of the guidelines will significantly lower clinical contribution of S100B sampling. The main reason for being classified in the “direct CT” groups in our study was age ≥ 65 and anti-platelet medication and our population was on average almost 10 years older than the population studied retrospectively by Undén et al. [13]. AHUS is located in close proximity to Oslo University Hospital, which is responsible for the management of the most severe traumas. The population attending the ED of AHUS is therefore dominated by minimal and mild injuries, intoxicated patients and elderly with moderate injuries. The patients being older in average could explain both the higher percentage of patients in the “direct CT” group and the higher prevalence of positive CT scans. One might argue that the guidelines therefore should only be applied for the population between 18 and 65 years only, but a recent study did find a reasonable specificity of 18.7% in patients aged 65 or older which supports the rationale for keeping the 65+ included in the guidelines [18].

Still the clinical usefulness of S100B is likely to be higher for the younger age groups. Applying the guidelines for MTHI patients under the age of 18 is therefore tempting, but due to large variation in the normal values for S100B in the younger age group, this is still not recommended by the SNC group [19].

As shown in a recent study, the clinicians should keep in mind that the S100B levels might be affected by the patient’s ethnicity, with especially African Americans showing significant deviations in S100B levels compared to Caucasians [20]. As the population of this study and the patient population of AHUS consist of 99% Caucasians, we do not think that this will significantly influence our results.

As previously mentioned, the clinical usefulness of S100B also depends on the time it takes from the clinical evaluation to the presence of a test result, which in the case of a positive S100B test will be the same as a delay in having an indicated CT examination. A near 2-h delay, as was the case in this study, for diagnosis and management of a traumatic brain haemorrhage may in some cases have severe or even fatal consequences. However, no such incidents were found in this study nor other previous studies examining the use of S100B as a marker for brain injury [9, 11, 17]. Further on, it is recommended that the patients are observed while waiting for the S100B results, and deterioration in GCS at this time will indicate an acute CT examination.

Nevertheless, when the S100B-test is used, there will be a near 2-h delay before the results are ready, and if the test is positive and a CT examination has to be supplemented, approximately three and a half hours would have been spent from the time the S100B-test was requisitioned until the CT examination can be analysed. This could affect the willingness to comply with the guidelines and has the potential to create insecurity both for the patient and the healthcare workers involved.

The main reason to introduce these guidelines was to reduce the number of unnecessary CT scans, and thereby reduce the costs and the radiation exposure to the patients. The estimated number of CTs that will lead to the development of a cancer vary depending on the specific type of CT examination, patient’s age and sex. A recent study estimated that 1 in 8.100 women who undergo a head CT will develop cancer from that CT, while the number was 1 in 11.080 men. For 20-year olds the risks were approximately doubled, and for 60-year olds, the risk was approximately 50% lower [21]. Avoiding up to 20% of the CT scans with optimal compliance might therefore make a substantial change for the MTHI patients.

As to the costs of a CT scanning, they will include the using of the machine itself and the wages of the staff who perform the scans and analyse the pictures. The costs do also vary between countries and are therefore hard to estimate. A study from the United States showed average price for upper-tier academic hospitals $1390.12 ± $686.13 [22], which means that AHUS, with a prize of about 14 USD per S100B analysis, throughout a year will save a six figure number in US dollars with the introduction of these guidelines.

In conclusion the clinical usefulness of the new guidelines and S100B in particular, depends on both the characteristics of the patient population and the compliance with the guidelines. Further efforts need to focus on increasing the adherence to a negative test result and avoid the “just in case” blood samples or CT scans. The responsibility must be put on the doctors who refer the patients to CT and we believe that education, information and experience with both the guidelines and the S100B test in particular, would increase the compliance within this group and consequently increase the clinical value of updated Scandinavian guidelines for initial management of minimal, mild and moderate head injuries.

### Limitations

The main limitation in this study is that the compliance of the guidelines was assessed more or less during the introduction period of these guidelines. This was partly tactical believing that a study itself will increase the focus on the new guidelines and facilitate the introduction. Secondly, in the study period a study form was included for each MTHI patient containing information of the new guidelines with check boxes to fill in, which to some degree dictated the pathway in the guidelines. It is also likely that there will be some overuse of S100B in the introduction period just to “get familiar” with the test which might be reduced and stabilized by time. The long-term compliance in an “unsupervised” setting is therefore unknown. Uncertainty of the time of injury could represent a source of error in the interpretation of the S100B, but the information is considered reliable as a big majority of the patients attend the ER directly from the site of trauma, many of them by ambulance, and because of the short distances in the catchment area.

## Conclusion

The implementation of the S100B and the updated SNC guidelines resulted in one third of the MTHI cases being discharged without further observation or CT examinations. One in five of these discharges was a direct result of S100B testing. However, almost half of the negative S100B results were ignored and in total the guidelines were not followed for nearly 40%. Nevertheless, no readmissions or missed severe traumatic injuries to the brain were observed.

## Abbreviations

AHUS: Akershus University Hospital
CT: Computer tomography
EPR: Electronic patient records
GCS: Glasgow Coma Scale
MTHI: Minimal, mild and moderate traumatic head injury
SNC: Scandinavian Neurotrauma Committee
